# Posttransplantation clonal dynamics of hematopoietic stem cells carrying prenatal and early‐life *DNMT3A* mutations

**DOI:** 10.1002/hem3.70262

**Published:** 2025-12-09

**Authors:** Lucca L. M. Derks, Konradin F. Müskens, Markus J. van Roosmalen, Aniek O. de Graaf, Nina Epskamp, Laurianne Trabut, Rico Hagelaar, Joop H. Jansen, Caroline A. Lindemans, Maaike G. J. M. van Bergen, Ruben van Boxtel, Mirjam E. Belderbos

**Affiliations:** ^1^ Princess Máxima Center for Pediatric Oncology Utrecht The Netherlands; ^2^ Oncode Institute Utrecht The Netherlands; ^3^ Laboratory of Hematology Radboud University Medical Center Nijmegen The Netherlands; ^4^ Wilhemina Children's Hospital Utrecht The Netherlands

## Abstract

Clonal hematopoiesis (CH), a prevalent and premalignant state in the elderly, has been detected in young individuals under selective pressures such as hematopoietic cell transplantation (HCT). However, the origin of CH and mutational processes underlying CH driver mutations in young blood systems remain unclear. Here, we used genome‐wide somatic mutation profiles to retrospectively trace the origin of *DNMT3A*‐mutant CH in three individuals, 14–41 years after childhood HCT. Both the rate and spectrum of somatic mutations in individuals with posttransplant CH were consistent with normal age‐associated mutagenesis. Phylogenetic analysis revealed that *DNMT3A*‐mutant HSPCs were present in the donor before 6.8 years of age, including during fetal development, despite being undetectable with a limit of detection of variant allele frequency of 0.001 at the time of transplantation. These findings were validated by comparing the observed mutations to expected age‐dependent mutational signatures. Our results reveal that undetectable *DNMT3A*‐mutant clones in young donors can expand into significant CH clones within decades upon transplantation. The rapid expansion of these clones in this context indicates that specific environmental pressures, rather than solely mutation acquisition, drive the development of CH.

## Graphical abstract

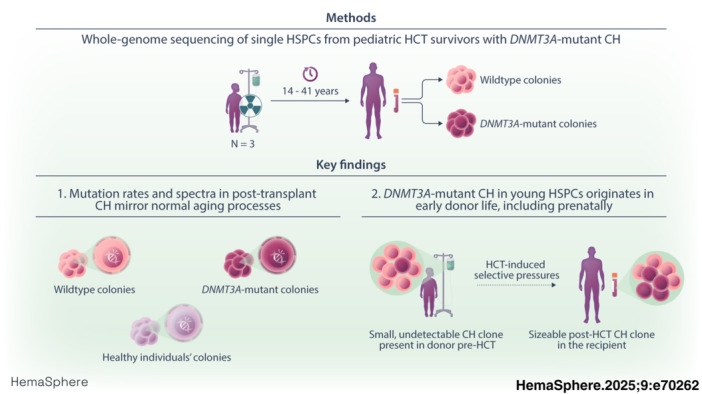

## INTRODUCTION

The life‐long production of mature blood cells is orchestrated by a hierarchy of hematopoietic stem and progenitor cells (HSPCs). As we age, HSPCs acquire mutations in their genomes at a rate of approximately 16 base substitutions and one small insertion/deletion (indel) per life year.^1^, ^2^, ^3^ While most of these mutations are evolutionary neutral, mutations in leukemia driver genes may affect HSPC expansion by providing a selective growth advantage under specific circumstances, resulting in clonal hematopoiesis (CH).^4^

CH is strongly age‐dependent and associated with an increased risk of developing myeloid malignancies, cardiovascular disease, and overall mortality.^5^, ^6^, ^7^, ^8^ While sizeable CH clones (i.e., with variant allele frequency [VAF] > 0.01) are rare below the age of 40, such clones are present in up to half of the general population aged 80 years or older.^5^, ^9^, ^10^ Previous studies suggest that CH driver mutations can be acquired early in life, followed by gradual clonal expansion.^11^, ^12^, ^13^ Notably, CH is more common in certain risk groups, including recipients of chemo‐ and radiotherapy treatment or hematopoietic cell transplantation (HCT).^14^, ^15^, ^16^, ^17^, ^18^ Previously, it has been demonstrated that after HCT, very large clones (VAF > 0.30) can be present in hematopoietic systems as young as 15 years.^15^ The presence of CH in young, transplanted blood systems raises the question of when and how these CH driver mutations arise.

Here, we study the genomes of single HSPCs from three childhood HCT recipients with CH, driven by a mutation in *DNMT3A*. We reveal that *DNMT3A* driver mutations are acquired in the donor very early in life, including prenatally. Despite being undetectable in the graft material, these drivers can result in large CH clones in a relatively short timeframe, under the selective pressures of HCT. Together, our findings emphasize the role of environmental pressures in the development of CH.

## MATERIALS AND METHODS

### Patient samples

Three HCT recipients with CH were selected from the 144 participants who enrolled in the original study.^15^ All participants provided written informed consent. This study was approved by the local ethics committee in accordance with the Declaration of Helsinki (NedMec no. NL77721.041.21). HCT recipients were selected based on young donor age and the presence of *DNMT3A*‐driven CH with a minimum VAF of 0.05 in peripheral blood to enable the comparison of both wildtype and mutant HSPCs. Historic DNA samples from the graft (available for two out of three study participants) were obtained from institutional biobank resources and HLA‐typing laboratories.

### CH measurement in historic DNA samples

CH was assessed in historic DNA samples using error‐correcting sequencing with single‐molecule inversion probes (smMIPs), targeting coding exons or selected regions of 27 myeloid and lymphoid driver genes, including *DNMT3A*, as described previously.^15^ Data processing and variant calling were performed with commercial analysis software (Sequence Pilot version 5.4.1; JSI Medical Systems). Loci that harbored the specific *DNMT3A* mutations in the long‐term follow‐up samples were manually inspected for the presence of mutant reads. The obtained sequencing coverage of >2900 reads per locus led to a detection threshold of VAF 0.001 for these specific mutations.

### Cell isolation and flow cytometry

Viably frozen peripheral blood mononuclear cells (PBMCs) were obtained via the biobank of the Princess Máxima Center for Pediatric Oncology. On the day of sorting, PMBCs were thawed in Iscove's modified Dulbecco's medium (Cat. No. 12440061; Thermo Fisher Scientific) supplemented with 20% fetal calf serum (Cat. No. F0804; Sigma‐Aldrich), followed by resuspension in Cell Staining Buffer (Cat. No. 420201; BioLegend) prior to sorting. Single live HSPCs were sorted on a SH800S cell sorter (Sony) using a 100um chip (Cat. No. LE‐C3210). Live HSPCs were defined based on the following markers: CD34+Lineage‐CD11c‐7AAD‐. Flow cytometry data were analyzed using the Sony SH800S Software (Sony).

### FACS antibodies

The following antibodies were obtained from BioLegend and were used for HSPC isolation: CD34‐BV421 (clone 561, 1:20; RRID AB_2561358); CD11c‐FITC (clone 3.9, 1:20; RRID AB_314173), Lineage (CD3/CD14/CD16/CD19/CD20/CD56)‐FITC (clones UCHT1, HCD14, 3G8, HIB19, 2H7, HCD56, 1:20; RRID AB_10612570). Viability staining solution 7‐amino‐actinomycin D (7AAD, 1:100, Cat. No. 420404) was also purchased from BioLegend. Propidium iodide (PI, 1:1000, Cat. No. P3566) was obtained from Thermo Fisher Scientific.

### Establishment of clonal HSPC cultures

Live (7AAD‐negative) Lineage‐/CD11c‐/CD34+ HSPCs were index‐sorted as single cells into flat‐bottom 384‐well plates (Cat. No. 781182; Greiner). Cells were cultured in StemSpan SFEM II medium (Cat. No. 09655; Stem Cell Technologies) supplemented with SCF (100 ng/mL, Cat. No. 130‐096‐692; Miltenyi); FLT3‐L (100 ng/mL, Cat. No. 130‐096‐474; Miltenyi); TPO (50 ng/mL, Cat. No. 130‐095‐745; Miltenyi); IL‐6 (20 ng/mL, Cat. No. 130‐093‐929; Miltenyi) and IL‐3 (10 ng/mL, Cat. No. 130‐093‐909; Miltenyi); UM729 (500 nM, Cat. No. 72332; Stem Cell Technologies) and StemRegenin‐1 (750 nM, Cat. No. 72342; Stem Cell Technologies); and primocin (100 μg/mL, Cat. No. ant‐pm‐1; InvivoGen). After 2–8 weeks of culture at 37°C and 5% CO_2_, expanded colonies were collected for DNA isolation and sequencing. Total DNA yield and time to colony harvest were used as surrogate markers for colony size and expansion rate, respectively. Due to a fungal infection in one plate, colonies that were harvested prematurely (from uninfected wells) were excluded from these analyses.

### Single‐cell whole genome amplification

Single live (PI or 7AAD‐negative) Lineage‐/CD11c‐/CD34+ HSPCs were index‐sorted and processed for whole‐genome amplification (WGA) using primary template‐directed amplification as described previously.^19^, ^20^ In brief, a single cell per well was sorted into LoBind PCR plates (Eppendorf, Cat. No. 0030129504) and stored at −80°C until processing. Cells were processed using the ResolveDNA® Whole Genome Amplification Kit v2.0 (Cat. No. 100545; BioSkryb) using a D100 single cell dispenser (HP).

### DNA isolation

Genomic DNA was extracted from clonal HSPC cultures using the QIAamp DNA Micro Kit (Cat. No. 56304; Qiagen) according to the manufacturer's instructions (Isolation of Genomic DNA from Small Volumes of Blood). DNA was eluted in 30 µL low EDTA TE buffer (10 mM Tris, 0.1 mM EDTA, Cat. No. 786‐150; G Biosciences) and measured using the Qubit dsDNA High Sensitivity kit (Cat. No. Q33231; Invitrogen).

### Genotyping of expanded colonies and whole‐genome amplified single cells

The presence or absence of the *DNMT3A CH* variants was assessed by Sanger sequencing (Macrogen) on DNA isolated from expanded colonies and single HSPCs directly processed for WGA. Each locus was amplified by PCR (Phusion™ High‐Fidelity DNA Polymerase, Cat No. M0530L; New England Biolabs), using the primers listed in Supporting Information S1: Table S1. For single‐cell genotyping, the number of false negative genotyping calls was extrapolated from the frequency of homozygous mutant cells.

### Whole genome sequencing (WGS) and read mapping

15–50 ng of DNA was used per sample to generate Illumina sequencing libraries using standard protocols. Each library was sequenced on an Illumina Novaseq. 6000 system (2 × 150 bp) to a base coverage of ×15. The sequencing reads were mapped to the human reference genome GRCh38 using the mapping tool Burrows‐Wheeler Aligner v0.7.a with settings: “bwa mem ‐c 100 ‐M.”^21^ Duplicate sequence reads were flagged using Sambamba (v0.6.8).^22^ GATK tools BaseRecalibrationTable and BaseRecalibration (v.4.1.3.0) were used to perform base recalibration.^23^ As a matched germline sample from the hematopoietic cell donor was unavailable, we created a single pseudo‐bulk germline reference sample with ×30 base coverage by merging two clonally unrelated HSPCs (sequenced to ×15 base coverage) using samtools (v.1.3.1).^24^ We randomly selected two sets of distinct pairs of HSPCs that were wildtype for *DNMT3A* or, if unavailable, HSPCs that did not harbor a *DNMT3A* driver mutation shared with another HSPC. All remaining analyses in this manuscript were repeated for both sets of HSPCs to confirm that the results were independent of the selected germline reference pseudo‐bulk. A full description of the pipeline is available at: https://github.com/ToolsVanBox/ASAP (version used: 1.0.1).

### Calling, filtration, and annotation of mutations

We performed mutation calling on all samples of a single individual in parallel using GATK HaploTypeCaller.^25^ All GATK tools used were part of version 4.1.3.0. Variants in the resulting multi‐sample VCFs were filtered by GATK SelectVariant with parameters “*select_type* = *type* =* ‘SNP’? ‘–select‐type SNP –select‐type NO_VARIATION’: ‘–select‐type INDEL –select‐type MIXED’.*” Next, GATK VariantFiltration was run with the following options: *–filter‐expression “MQ* < *40.0” –filter‐expression “FS* > *60.0” –filter‐expression “HaplotypeScore* > *13.0” –filter‐expression “MQRankSum <−12.5” –filter‐expression “ReadPosRankSum <−8.0” –filter‐expression “MQ0* > = *4* & ((MQ0/(1.0* DP)) > *0.1)” –filter‐expression “DP* < *5” –filter‐expression “QUAL* < *30” –filter‐expression “QUAL* > = *30.0* & *QUAL* < *50.0” –filter‐expression “SOR* > *4.0” –filter‐name “SNP_LowQualityDepth” –filter‐name “SNP_MappingQuality” –filter‐name “SNP_StrandBias” –filter‐name “SNP_HaplotypeScoreHigh” –filter‐name “SNP_MQRankSumLow” –filter‐name “SNP_ReadPosRankSumLow” –filter‐name “SNP_HardToValidate” –filter‐name “SNP_LowCoverage” –filter‐name “SNP_VeryLowQual” –filter‐name “SNP_LowQual” –filter‐name “SNP_SOR” ‐cluster 3 ‐window 10 –filter‐expression “QD* < *2.0.*” The remaining variants were annotated using SNPEffFilter,^26^ SNPSiftDbnsfp (dbNSFP3.2a),^27^ GATK VariantAnnotator (COSMIC v.89), and SNPSiftAnnotate (GoNL release 5). The steps in this section are part of the pipeline available at: https://github.com/ToolsVanBox/ASAP (version used: 1.0.1).

### Filtering of germline and subclonal variants

We obtained a high‐quality catalog of somatic mutations through additional post‐processing filtering steps as described.^28^, ^29^, ^30^ In brief, we selected high‐quality variants with (1) a base coverage of at least 5, (2) a GATK phred‐scaled quality score R 100, (3) had a mapping quality of 55 or higher, (4) a GATK genotype quality of 99 or 10 (for heterozygous and homozygous variants, respectively) in both the pseudo‐bulk reference and the sample of interest. We removed variants with a VAF below 0.15 to exclude technical artifacts and variants that arose during the clonal expansion of the parental HSPC of each colony in vitro. Finally, germline variants were removed by excluding all variants with any evidence in the pseudo‐bulk control sample. Since donor/recipient chimerism can lead to incorrect germline filtering, for each HCT recipient, we used Picard CrosscheckFingerprints (GATK)^23^ to confirm that all *DNMT3A*‐wildtype colonies originated from the same individual as the CH, which was previously established to originate from a donor HSPC.^15^ A full description of the pipeline is available at: https://github.com/ToolsVanBox/ASAP (versions used: 1.0.1/1.0.3).

### Estimation of filtered shared fetal mutations

The germline filtering was performed with a pseudo‐bulk sample comprised of two clonally unrelated HSPC colonies to minimize the shared ancestral origin between the pseudo‐bulk and the single colonies. This approach leads to a small underestimation of the somatic mutation load, as random pairs of HSPCs are expected to share several somatic variants due to mutation accumulation early in development.^31^, ^32^ To model the number of mutations that is missed by performing germline filtering with randomly sampled HSPCs, we used previously published phylogenetic trees to calculate the number of overlapping variants between HSPCs.^32^ For both trees, we randomly sampled three tips (one “sample of interest” and two “samples in pseudo‐bulk”) a thousand times using the R packages base (v. 4.4.0)^33^ and ape (v. 5.8‐1).^34^ All edges connecting the most recent common ancestor of a pseudo‐bulk sample and the sample of interest to the root were retrieved. Finally, we calculated the number of shared variants as the sum of the edge lengths of all unique edges identified for either of the two pseudo‐bulk samples. The median number of shared variants was determined for all thousand iterations of randomly sampled HSPCs per tree/fetus. Finally, the mean of the results per fetus was used as the final number of shared fetal mutations.

### Somatic autosomal mutation load

To compare the somatic mutation load in the recipient's HSPCs to a healthy baseline, we applied the analysis pipeline described above to previously published samples of healthy donors.^1^, ^2^ The BAM files of non‐germline samples were subsampled to ×15 base coverage to conform with the sequencing coverage of the samples included in this manuscript. The total number of autosomal mutations was corrected for the callable genome fraction, defined as the sum of the intersect of GATK's CallableLoci (v. 4.1.3.0) “CALLABLE” regions in each sample and its matched pseudo‐bulk reference, normalized to the autosomal callable genome size (2745186691). The healthy control samples were used to construct a mixed‐effects model for the mutation load by age, taking donor dependency into account, using the R package lme4 (v.1.1‐35.5).^1^, ^35^ For each HCT recipient in this study, the expected mutation load was determined using the slope and intercept of the mixed‐effects model and the age of the recipient at the time of sampling. Finally, the total number of observed autosomal variants, with the addition of the number of estimated shared fetal variants, was corrected for the callable genome fraction and the expected mutation load. The corrected mutation loads were compared by a Wilcoxon rank sum test (two‐sided, Bonferroni corrected) using the R package rstatix (v.0.7.2),^36^ and visualized using the R package ggpubr (v.0.6.0).^37^ This analysis was performed for two independent sets of germline reference samples, to validate that any shared variants between the studied HSPCs and the two randomly sampled HSPCs do not affect the final outcome (see “Filtering of germline and subclonal variants” for details on germline filtering).

### Mutational signatures in *DNMT3A*‐wildtype and mutant HSPCs

We performed de novo signature extraction on all unique somatic single‐base substitutions identified in the recipient HSPCs based on nonnegative matrix factorization (NMF) using the R package MutationalPatterns (v.3.16.0),^38^ combined with BSgenome reference genome ‘BSgenome.Hsapiens.NCBI.GRCh38’.^39^ To enable robust signature extraction of clock‐like signatures SBS1, SBS5, and HSPC, as well as SBS18, which was identified in HSPCs,^2^ we included single base substitutions of previously published datasets of healthy adult and pediatric tissues.^1^, ^40^ The extraction was performed using the *extract_signatures* function with parameters “nrank = 4, nrun = 100.” Rank 4 was chosen based on the inflection point between the input matrix and the corresponding estimate. Next, we replaced the extracted signatures with their most similar COSMIC database v3.2 or HSPC/SBSblood.^41^ counterpart, with a minimum cosine similarity of 0.85. As individual HSPC‐derived colonies part of the CH shared a clonal origin, only unique mutations per genotype (*DNMT3A*‐wildtype or mutant) were included for the signature refit to prevent the use of individual mutations multiple times. The refitting was performed with the extracted signatures using *fit_to_signatures_bootstrapped* with parameters “method = ‘strict’, n_boots = 100” and subsequent averaging of all iterations. To validate that the number of signatures extracted through NMF was chosen correctly, the cosine similarity of the original versus reconstructed profiles was plotted using *plot_original_vs_reconstructed*.

### Construction of phylogenetic lineage trees

As input for phylogeny inference, we used the multisample VCF output of GATK HaploTypeCaller, filtered for the positions of somatic autosomal variants. Phylogeny reconstruction of the HSPC colonies was performed using a maximum likelihood framework that allows for allelic dropout using CellPhy (v.0.9.2),^42^ which incorporates RAxML‐NG (v.0.9.0).^43^ We ran CellPhy on the phred‐scaled genotype‐likelihood values using the GT16+F0 model with 100 bootstrap iterations. Next, the support for each split in the tree was determined using CellPhy's “‐‐support” function, and the mutations were mapped to the tree using CellPhy's “‐‐mutmap” function (with setting “opt‐branches off”). Only splits with presence in all iterations were considered for timing. The resulting trees were visualized using ggtree (v. 3.8.2).^44^ Each tree was rooted using the pseudo‐bulk sample as output using the *root* function from the R package ape (v.5.8).^34^ A wrapper for the full reconstruction as described above is available at https://github.com/ToolsVanBox/CellPhyWrapper. The wrapper includes steps to remove mutations from end branches if read support for this variant is present in multiple samples. In addition, mutations that are assigned to a branch, but are not present in all descendant samples, are reassigned to the best‐fitting branch. Here, a cutoff of 0.1 is used for the fraction of samples in which a shared mutation can be missing (e.g., due to low sequencing coverage).

The scale of each tree was converted from mutation‐time into time in years (“ultrametric”) using an iteratively reweighted means algorithm.^3^, ^11^ Previously published code^11^ was customized for compatibility with the output of CellPhy. The tree objects were loaded and converted using R packages ape (v.5.8‐1),^34^ treeio (v.1.30.0, to retrieve tips)^45^ and phangorn (v.2.12.1, to retrieve node descendants).^46^ The conversion consisted of five steps: (1) the addition of filtered fetal variants, (2) correction for sequencing coverage, (3) splitting of the tree into a prenatal and postnatal region, (4) scaling of the prenatal and postnatal trees, and (5) merging of the scaled prenatal and postnatal trees. First, the previously estimated number of filtered fetal variants was added to the length of the root branch. Second, all branch lengths were corrected for sequencing coverage according to previously published methods,^12^ where the somatic variant sensitivity (*p*) equals the callable genome fraction as defined above. Private or end‐branches are scaled based on the genome coverage of the sample at its tip, whereas shared branches are scaled according to “1 – Π,” where Π is the product of “1 – *p*” for each sample descending from this shared branch. Third, the tree was traversed from the root to the tips and cut at the traveled distance corresponding to the mutation load at birth, which was derived from the baseline of mutation accumulation in HSPCs with age (Supporting Information S1: Figure S3B). Fourth, each part was linearly scaled to time in years using the iteratively reweighted means algorithm, where the ratio between the length of a shared branch to the average length of its descendant branches is retained.^3^, ^11^ The prenatal tree was scaled to 0.75 years, corresponding to a normal gestation period. The postnatal tree was scaled to the hematopoietic stem cell age, defined as the age of the donor at the time of HCT plus the length of time between the HCT and time of sampling of the recipient. Fifth, the scaled branches were combined into a single tree spanning the whole life of the HSPCs from conception to the time of sampling. Splits in the tree were timed by taking the total distance in years from birth (at 0.75 years) to the split. As mutation accumulation is linear only after birth, we calculated the uncertainty interval of these timings by using both the lower and the upper boundary of the 95% confidence interval of the mutation load at birth (52–121 mutations, retrieved using ggeffects (v2.0.0)^47^). Visualization was performed by using R packages ggtree (v.3.14.0),^44^ ggbreak (v.0.1.2),^48^ and in‐house plotting functions compatible with CellPhy (available at https://github.com/ToolsVanBox/CellPhyWrapper). The entire phylogenetic tree construction and conversion to time in years were performed twice for every HCT recipient, based on two independent sets of HSPCs used to filter germline variants (see “Filtering of germline and subclonal variants” for details on germline filtering).

### Mutation accumulation throughout the phylogenies

For every branch in the phylogenies, a single 96‐spectrum mutation matrix was created based on the mutations mapped to each branch using *extract_vcf_per_branch* and *convert_vcf_to_granges* (available at https://github.com/ToolsVanBox/CellPhyWrapper). For mutational signature analysis, the individual branches and the corresponding mutation matrices were merged into three classes (Figure 2A–C). The first class, termed “clonal,” was composed of branches that are shared between all *DNMT3A*‐mutant HSPCs of the same clone. This class represents all mutations present in the most recent common ancestor (MRCA) of the CH HSPCs, one of which is the *DNMT3A* driver mutation. The second class, termed “subclonal,” constitutes all branches descending from the clonal branch. This class is composed of all mutations that are acquired after the divergence of the *DNMT3A*‐mutant MRCA, all of which are accumulated after driver acquisition. The third class, termed “wildtype” or “WT,” consisted of all branches belonging to wildtype HSPCs. This class represents mutation accumulation in wildtype HSPCs. Branches belonging to HSPCs harboring unique *DNMT3A* mutations (i.e., found in a single colony) were left out of this analysis, as these branches cannot be divided into a clonal and subclonal fraction. Per recipient, every class was considered as an individual sample for mutation signature analysis, which was performed as described above for the wildtype and *DNMT3A*‐mutant genotypes.

To compare mutation accumulation throughout the phylogenies to mutation accumulation during aging, the cumulative mutational matrix up to each branchpoint in the tree was determined. We used a previously published method to predict the absolute base substitution spectrum at any given age in healthy individuals.^2^ For every branch, we calculated the cosine similarity between its cumulative mutation matrix and the predicted mutation matrix at every age (range 0–63).

### Probability of mutational signatures causing driver mutations

We employed methods similar to Morganella et al.^49^ to calculate the probabilities of the individual mutational signatures causing the *DNMT3A* driver mutations. Briefly, the contribution of each signature was multiplied by the fraction of the signature matching the trinucleotide context of the driver mutation. Only the signature contributions inferred from the “clonal” class of mutations as defined above were considered, as these represent the mutational processes active during the time interval of *DNMT3A* mutation acquisition. The probabilities were normalized to the sum of all probabilities per *DNMT3A* mutation.

### Statistical analysis

**Figure 1:**
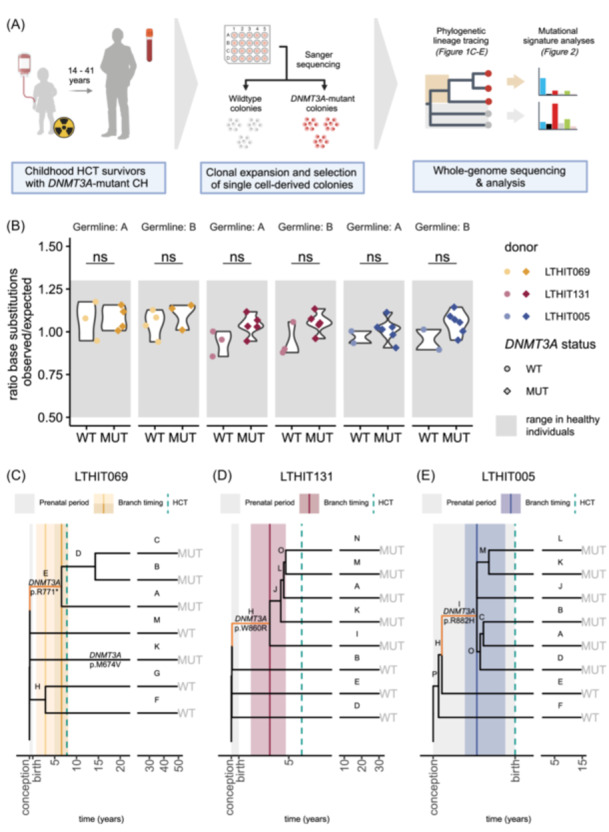
**Mutation accumulation in** ***DNMT3A‐*** **mutant CH mirrors normal aging and reveals early driver acquisition**. **(A)** Schematic overview of study setup. **(B)** Somatic autosomal mutation loads of hematopoietic cell transplantation (HCT) recipient HSPCs, normalized to the expected mutation load by hematopoietic age. The mutation loads were determined in two parallel analyses using distinct pairs of HSPCs for germline filtering (A and B). Comparison of mutation loads was performed by Bonferroni‐corrected two‐sided Wilcoxon rank sum tests (LTHIT069 *P* = 1 (A and B), LTHIT131: *P* = 0.86 (A) and 1 (B), LTHIT005: *P* = 1 (A and B). WT, *DNMT3A*‐wildtype. MUT, *DNMT3A*‐mutant. **(C)** Time‐scaled phylogenetic tree of single HSPC colonies of LTHIT069, including *DNMT3A*‐wildtype (WT) and mutant (MUT) colonies, obtained after inference by *n* = 100 bootstrap iterations using a maximum likelihood framework. The *DNMT3A* variants, including the corresponding amino acid change, are labeled to distinguish between distinct mutations. *DNMT3A* p. I705T was not detected in in vitro expanded HSPCs. Vertical dashed line (green) represents the time of HCT. Vertical solid lines and shaded interval (yellow) indicate the time estimate and uncertainty interval of the branchpoint, respectively. **(D)** Similar to (C), but for LTHIT131 with time estimates in red. **(E)** Similar to (C), but for LTHIT005 with time estimates in blue.

The number of cells analyzed per genotype per HCT recipient is shown as individual datapoints in Figure 1B, excluding the two clonally unrelated HSPC colonies that were merged into a pseudo‐bulk reference sample for germline filtering. *P*‐values were calculated using a two‐sided Wilcoxon rank sum test with Bonferroni correction for multiple testing unless noted otherwise and shown in the figure legends. The effect of *DNMT3A* mutation status on growth characteristics (time to harvest and total DNA yield) was tested using linear mixed‐effects models (lme4 and lmerTest packages^35^, ^50^), with mutation status as a fixed effect and patient as a random intercept. The expected and observed fractions of mutant cells in colonies and genotyped HSPCs were compared using two‐sided binomial tests, and uncorrected *P*‐values are shown. Bootstrapped analyses are based on 100 iterations, as noted in the figure legends. Unless stated otherwise, all data visualizations are performed with the R package *ggplot2* (v.3.5.1),^51^ obtained as part of the *tidyverse* suite of packages.^52^

## RESULTS

### Mutation accumulation in posttransplant CH is consistent with normal aging

To investigate the rate and spectrum of somatic mutation accumulation in CH decades after childhood HCT, we performed WGS on clonally expanded HSPCs (Figure 1A).^1^, ^2^, ^41^ We obtained samples from three childhood HCT recipients with a follow‐up period of 14–41 years, recruited in a previous study on posttransplant CH (Table 1).^15^ In each case, the recipient harbored at least one *DNMT3A*‐mutant clone (VAF range of the largest clone: 0.07–0.31). The recipients were transplanted for hematological malignancies after myeloablative conditioning, using stem cells from bone marrow (*n* = 2, age 6 and 7 years) or umbilical cord blood (*n* = 1). Per recipient, we performed WGS on 9–10 single CD34+ HSPCs derived from peripheral blood after clonal expansion in vitro, hereafter referred to as colonies. Of these, 4–5 wildtype and 4–6 *DNMT3A‐*mutant colonies were selected based on Sanger sequencing of the previously identified *DNMT3A* variants.^15^ For LTHIT069, in whom multiple expanded *DNMT3A‐*mutant lineages were identified previously,^15^ only two out of three variants were detected in the in vitro expanded colonies. We validated that all the assessed colonies were donor‐derived (Methods). *DNMT3A*‐mutant colonies were observed at higher frequencies than expected based on the VAF in peripheral blood (Supporting Information S1: Figure S1A, combined binomial test *P* = 0.03), especially when carrying the *DNMT3A* p.R882H variant. The increased frequency of mutant colonies could be caused by an increased capacity of mutant cells to grow out in vitro, or by a larger proportion of mutant cells in the HSPC compartment compared to the peripheral blood. In line with the first explanation, we observed that *DNMT3A*‐mutant colonies grew faster and larger compared to wildtype colonies, based on the time until harvest and DNA yield (Supporting Information S1: Figure S1B–E, linear mixed model *P* = 0.038 and 0.015, respectively). To investigate if the proportion of mutant cells in the HSPC compartment is larger compared to peripheral blood, we performed single‐cell genotyping (Methods)^19^, ^20^ to directly determine the fraction of HSPCs carrying the *DNMT3A* driver mutations. This approach excludes any potential bias introduced by in vitro expansion. The proportion of single‐cell genotyped mutant HSPCs aligned closely to the expected proportion based on peripheral blood VAF for LTHIT005 (expected: 0.60, observed: 0.64, binomial test: *P* = 0.56) and LTHIT131 (expected: 0.62, observed: 0.60, binomial test: *P* = 1.0 Supporting Information S1: Figure S2A). This indicates that the proportion of mutant cells in the HSPCs is equal to that in peripheral blood, further supporting the finding that *DNMT3A*‐mutant cells have a clonal outgrowth advantage in vitro. For LTHIT069, only four HSPCs were successfully genotyped, preventing a reliable estimation of mutation frequencies in the HSPC compartment. Notably, one HSPC with both p.R771* and p.I705T variants was observed, indicating that the p.I705T variant is a subclone of p.R771* (Supporting Information S1: Figure S2B). To identify the somatic mutations in the parental HSPC of each colony, subclonal and germline variants were filtered using randomly sampled HSPCs as germline reference (Methods).^1^, ^29^ We estimated that our germline filtration approach resulted in the removal of six true somatic mutations, for which we corrected the total mutation counts (Methods, Supporting Information S1: Figure S3A). To compare autosomal mutation loads to mutation accumulation in normal aging, we applied the variant calling and filtration pipelines of this study to previously published samples of healthy individuals (Supporting Information S1: Figure S3B).^1^, ^2^ The mutation burden of all recipient HSPCs was within the range observed during normal aging (observed over expected ratios: 0.9–1.2, normal aging range: 0.5–1.3, respectively, Figure 1B). We confirmed that the use of independent sets of germline reference samples yielded identical results, indicating that the germline reference samples used were phylogenetically unrelated to the remainder of the samples (Supporting Information S1: Figure S4A,B). Importantly, the number of somatic mutations was similar between *DNMT3A*‐mutant and wildtype HSPCs (Wilcoxon test with Bonferroni correction, *P* > 0.05, Figure 1B), showing that mutant *DNMT3A*‐driven clonal expansion after childhood HCT is not associated with increased mutagenesis, even decades after HCT.

**Table 1:** Clinical data for research participants.

**General information**					
Sample name	LTHIT005	LTHIT131	LTHIT069		
Recipient age at study (years)*	30	26	42		
HSC age at study (years)	14.5	30	49.1		
Sex	Male	Female	Female		
Material used for CH measurement	Whole blood	Whole blood	Whole blood		
CH	Yes	Yes	Yes		
**HCT characteristics**					
Disease category	Hematologic malignancy	Hematologic malignancy	Hematologic malignancy		
Stem cell source	Cord blood	Bone marrow	Bone marrow		
Recipient age at HCT (years)^a^	16	3	0		
Donor age at stem cell donation (years)^a^	0	6	7		
Follow‐up time (years)^a^	14	23	41		
Chimerism at study (donor contribution)	97%	100%	100%		
**CH driver mutations**					
Mutation no.	LTHIT005‐1	LTHIT131‐1	LTHIT069‐1	LTHIT069‐2	LTHIT069‐3
Gene	*DNMT3A*	*DNMT3A*	*DNMT3A*	*DNMT3A*	*DNMT3A*
c.HGVS	2645G>A	2578T>C	2311C>T	2020A>G	2114T>C
p.HGVS	Arg882His	Trp860Arg	Arg771Ter	Met674Val	Ile705Thr
Variant allele frequency	0.30	0.31	0.073	0.029	0.025
Mutant reads	915	836	243	60	94

^a^ Rounded down to the first integer.

The mutagenic processes underlying mutation accumulation leave characteristic mutational signatures.^53^ The HPSCs studied here had a very low mutational background at time of donation, due to the young age of the donors (including a neonatal donor), and were studied decades after HCT. Therefore, the mutational spectrum is dominated by somatic mutations acquired posttransplantation, increasing the sensitivity of detecting altered mutagenesis posttransplantation. We performed signature extraction and refitting as described previously (Methods).^1^, ^2^ The endogenous, clock‐like signatures SBS1, SBS5, and HSPC/SBSblood were found to predominate in both *DNMT3A*‐mutant and wildtype HSPCs (Supporting Information S1: Figures S1C,S5A and S5B).^1^, ^41^ In line with the observation that the mutation load did not differ between wildtype and mutant HSPCs, the mutational spectra were similar between wildtype and mutant HSPCs in each HCT recipient (cosine similarities: LTHIT069: 0.99; LTHIT131: 0.98; LTHIT005: 0.93). These findings align with previous studies on healthy individuals^3^ allogeneic HCT recipients^12^, ^54^ and gene therapy recipients,^55^ as well as *DNMT3A*‐mutant blood‐derived lymphoblastoid cell lines and bulk AML samples.^56^, ^57^ Overall, our results indicate that the rate and spectrum of mutation accumulation in *DNMT3A*‐mutant CH after childhood HCT are comparable to those observed during healthy aging.

### Driver mutations for clonal hematopoiesis are acquired in pediatric donors prior to HCT

To retrospectively trace the acquisition of the CH‐associated *DNMT3A* mutations in the blood of childhood HCT recipients, we constructed phylogenies for each recipient based on a maximum likelihood framework (Methods).^42^ As somatic mutations accumulate gradually throughout life and are propagated to the progeny of a shared ancestral cell, they can be used to assess the clonal composition of tissues.^1^, ^41^ We confirmed that the wildtype HSPCs generally shared only 0–10 somatic mutations before branching into individual lineages, characteristic of the very polyclonal origin of blood (Figure 1B–D)^3^, ^32^ In one individual, two HSPCs without CH drivers showed a common origin, sharing 128 somatic variants before branching into separate lineages (Figure 1B). This finding is not unexpected, as clade expansion without apparent known driver mutations has been described in individuals with and without prior HCT.^3^, ^11^, ^12^ In contrast to the wildtype HSPCs, every colony that harbored the same *DNMT3A* variant shared a clonal origin (Figure 1B–D). Based on the point of branching into separate lineages of the *DNMT3A*‐mutant HSPCs, which reflects an ancestral cell that divided in the past, we inferred the latest time point at which a CH driver mutation was acquired. Given that *DNMT3A*‐mutant HSPCs accumulate somatic mutations in a linear fashion throughout postnatal life at the same rate as HSPCs in aging individuals (Figure 1B), we scaled the trees to linear time using an iteratively reweighted means approach (Methods).^3^, ^11^, ^12^ We first excluded a potential bias in the tree construction due to our germline filtering approach, which can be introduced by shared variants between randomly sampled HSPCs, through tree construction and scaling to linear time for each HCT recipient using a second independent set of randomly sampled HSPCs (Supporting Information S1: Figure S4C–H). In all three HCT recipients, the estimated latest time of CH driver acquisition preceded the HCT procedure up to three years and was restricted to the first decade of life (LTHIT069: 6.8 y; LTHIT131: 3.1 y; LTHIT005: prenatal, Figure 1C–E, Supporting Information S1: Figure S4F–H). The pretransplant origin of the *DNMT3A* mutations was also observed when accounting for the variable mutation load at birth for two recipients, LTHIT131 and LTHIT005, as the entire uncertainty interval preceded the time of HCT (Figure 1D,E, Supporting Information S1: Figure S4G,H). For one HCT recipient (LTHIT069), the uncertainty interval overlapped with the time of HCT (5.1‐8.3 y), indicating that the driver acquisition occurred either before or soon after the time of the HCT procedure (Figure 1C and Supporting Information S1: Figure S4F). Notably, as LTHIT005 was transplanted with a cord blood donor, the *DNMT3A* variant must have arisen prenatally, during fetal development of the HCT donor. These findings validate results from a previous study on adult HCT recipients, showing that post‐transplant CH can be driven by *DNMT3A* mutations acquired early in the life of the donor, including prenatally.^12^ In contrast to these previous studies, where around a hundred colonies were sequenced per individual, we reconstructed the phylogenies of the CH based on a low number of HSPC colonies (±10). This indicates that our approach, which includes the genotyping of colonies prior to WGS, offers an opportunity to study the phylogenies of clonal lineages at reduced cost. Importantly, by studying a recipient of a cord blood graft, we demonstrate that postnatal expansion of mutant HSCs in the donor is not required for the development of posttransplant CH. Given the young age of the donors in our study, it is highly unlikely that these grafts already contained detectable CH. Indeed, for recipients from whom leftover graft material was available (LTHIT005 and LTHIT131), we confirmed the absence of detectable CH in the graft (detection threshold: VAF < 0.001, Supporting Information S1: Table S1). Therefore, the CH clones in these individuals expanded from being undetectable to a VAF of 0.30 and 0.31, within 14 and 23 years, respectively. Together, these data indicate that *DNMT3A* mutations, which are generally associated with age‐related CH, can already be acquired very early in life, including prenatally, and transmitted upon transplantation to a foreign recipient, followed by expansion to significant clones within 14–23 years.

### Mutational signature analysis supports early driver acquisition

**Figure 2:**
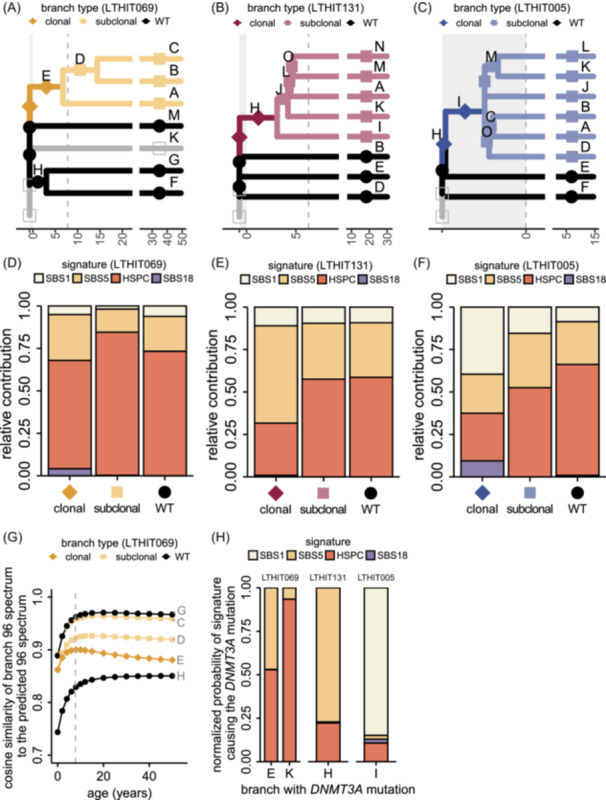
**Mutational signature analysis supports early and prenatal acquisition of** ***DNMT3A*** **driver mutations**. **(A)** Class annotation of individual branches and their underlying somatic mutations in the phylogenetic tree of LTHIT069. Classes: clonal (diamond), subclonal (square, filled), and wildtype (circle). Open squares indicate branchpoints without underlying somatic mutations or branches related to HSPC colonies with non‐recurrent *DNMT3A* mutations. Dashed vertical line (gray) represents the time of HCT. **(B)** Similar to (A), but for LTHIT131. **(C)** Similar to (A), but for LTHIT005. **(D)** Refit of mutational signatures to the mutation classes for LTHIT069 as in (A), showing relative contributions per signature. The contributions were obtained through bootstrapped refitting (*n* = 100) of the signatures extracted by nonnegative matrix factorization. **(E)** Similar to (D), but for LTHIT131 as in (B). **(F)** Similar to (D), but for LTHIT005 as in (C). **(G)** Cosine similarities between the cumulative mutation matrix of a single branch and the predicted mutation matrix at any given age, for the phylogeny or LTHIT069. Representative branches are shown for *DNMT3A*‐wildtype branches (circles) and subclonal branches (squares). **(H)** The probabilities of individual *DNMT3A* mutations being caused by the identified mutational signatures. *DNMT3A* mutations are indicated by their location in a branch in the phylogeny.

Mutational signature analysis provides an additional opportunity to validate the acquisition of CH drivers early in the life of the donors. During prenatal development and early life, the pattern of mutation accumulation is distinct from adult life.^2^, ^31^ The mutational spectrum in neonatal HSPCs is dominated by patterns SBS1 and SBS5, with minor contributions of SBS18 and the HSPC/SBSblood signature. In postnatal HSPCs, the HSPC signature becomes predominant (Supporting Information S1: Figure S3C).^2^ To validate the phylogenetic analyses, we divided the somatic mutations underlying the topology of each tree into separate classes (Methods, Figure 2A–C). Per HCT recipient, we refitted the signatures present in the HSPCs to each class of mutations (Figure 2D–F, Supporting Information S1: Figure S5C–E). In LTHIT069, the clonal branch diverged at approximately 7 years of age, and as expected, the HSPC signature is predominant (Figure 2A,D).^2^ The clonal branch in recipient LTHIT131 separated into individual lineages below 5 years of age and this is reflected in its mutational patterns; SBS1 and SBS5 together make up a larger fraction of the mutational spectrum than the HSPC signature, which becomes more predominant in the subclonal branches (Figure 2B,E). The prenatal origin of the clonal branch in LTHIT005 is supported by the large contribution of SBS1 (Figure 2C,F). However, the total number of mutations in this branch is lower than 50, leading to a less accurate refit in the clonal class (Supporting Information S1: Figure S5D).

For a more detailed analysis of age‐related mutation accumulation, we compared the sum of accumulated mutations up to specific points in the phylogenies to the modeled mutational spectrum for each year of life in healthy human HSPCs.^2^ First, we confirmed that the terminal branches showed a mutational spectrum according to the expected patterns at the time of sampling. In all three HCT recipients, the terminal branches reached a plateau in cosine similarity (>0.95) to the predicted spectrum later in life (Figure 2G and Supporting Information S1: Figure S5F,G). Next, we investigated the mutational spectrum of the internal branches, taking into account the estimated stem cell age at which the branch diverged. Here, we observed different outcomes for very early branchpoints versus branchpoints later in life. The mutational spectra of the branches that diverged very early in life did not closely resemble the expected mutational spectrum at any age (cosine similarity <0.9, Supporting Information S1: Figure S5F,G), potentially due to the very low mutation numbers accumulated thus far in life. In contrast, the mutational spectrum of the clonal branch of LTHIT069 was most similar to the expected spectrum in the age range surrounding the HCT (cosine similarity>0.9, Figure 2G). This finding corresponds to the predicted time of divergence of the clonal branch based on the phylogenetic analyses. A similar phenomenon was observed for subclonal branch D, which reaches its highest cosine similarity at a higher predicted age than the clonal branch E (Figure 2G). Overall, despite challenges related to low mutation numbers, mutational signature analysis supports the time estimates based on the phylogenetic analyses.

Finally, we estimated the probability of different substitution signatures to cause the CH driver mutations based on the trinucleotide context of the mutation and the activity of the signature in the clonal branch (Figure 2H).^49^ For recipients LTHIT069 and LTHIT131, the *DNMT3A* mutations are most likely caused by the mutational processes underlying SBS5 and the HSPC signature (LTHIT069: 47% and 53% probability, respectively. LTHIT131: 77% and 22% probability, respectively). This corresponds to the estimated postnatal origin of these mutations (Figure 1C,D). The CH driver in recipient LTHIT005, on the other hand, is very likely caused by SBS1 (85% probability). As SBS1 is most active in HSPCs during prenatal development (Supporting Information S1: Figure S3C),^2^, ^31^ this further supports the prenatal origin of the *DNMT3A* mutation in LTHIT005. In conclusion, both the spectrum of mutations in the clonal branches and the likely causative processes behind the CH driver mutations correspond with the early acquisition of *DNMT3A* mutations, prior to HCT, as early as prenatal development.

## DISCUSSION

Here, we demonstrate that mutant *DNMT3A‐*driven CH at a young hematopoietic age is driven by mutations acquired early in life, including prenatally. The mutational signatures most likely inducing these driver mutations match the dominant mutational signatures at the time of driver acquisition. Our data confirm and extend previous findings that suggest the early—and potentially prenatal—origin of *DNMT3A* mutations in CH.^11^, ^12^, ^13^ Finally, we show that transplanted *DNMT3A*‐mutant clones can expand from being undetectable (VAF < 0.001) at the time of HCT to dominating the HSPC compartment within decades, highlighting the importance of external pressures in the development of CH.

Accurate timing of the time of driver acquisition through phylogenetic analyses is based on the assumption that the rate of mutation accumulation is constant throughout the rise of CH.^12^ Here, we justify this approach through comparison of CH HSPCs, their matched wildtype counterparts, and HSPCs from healthy individuals. Our results align with results from other studies in HCT and gene therapy cohorts, showing highly similar mutation burdens in matched donors and recipients.^12^, ^54^, ^55^ Thus, even in individuals who develop CH decades after childhood HCT, mutation accumulation before and during clonal expansion corresponds to the rate and spectrum observed in normal aging, enabling accurate timing of *DNMT3A* mutation acquisition through phylogenetic analyses. Furthermore, we show that reconstructing clonal phylogenies is feasible with a limited selection of wildtype and mutant colonies prior to WGS. This substantially reduces the resources needed and increases the accessibility of retrospective lineage tracing studies based on somatic mutations.

Unlike prior studies of adult HCT donors and recipients,^12^ our work focuses on recipients of HCT in childhood. In general, donor age correlates with the risk of CH development after HCT and gene therapy.^15^, ^55^ Since young stem cells have a lower mutation burden,^29^ it has been proposed that using young stem cell products may minimize the risk of therapy‐induced clonal expansions.^55^ Yet, we observed that the transplantation, engraftment, and expansion of HSPCs with pre‐existing, but undetectable *DNMT3A* mutations can occur even when transplanting neonatal material. Thus, while young donor age may lower CH risk, it does not fully eliminate the potential presence of pre‐existing mutant HSPCs that may expand upon HCT. The prenatal origin of CH‐driving mutations in *DNMT3A* contrasts with the late detection of expanded *DNMT3A‐*mutant clones in aging individuals.^5^, ^6^ This may in part be explained by the requirement for environmental pressures to promote the expansion of mutant clones, with different conditions favoring different driver mutations.^18^ For example, *EZH2* mutations, also with potential prenatal origin, were selected more strongly than *DNMT3A* mutations after autologous HCT with genetically modified HSPCs,^55^ whereas lineages carrying *TP53* or *PPMD1* mutations, including a prenatally acquired *PPMD1* mutation, preferentially expanded upon exposure to chemotherapy.^58^ Together with our data, these findings suggest that environmental pressures such as HCT, rather than the timing of driver acquisition, are rate‐limiting for CH development.

While CH‐related driver mutations in genes other than *DNMT3A* have been shown to be donor‐derived,^12^, ^59^, ^60^ the very early acquisition as observed for *DNMT3A* mutations has not been recapitulated for other genes after allogeneic HCT.^12^ Future studies in both pediatric and adult HCT recipients may reveal whether the early or prenatal origin of driver mutations is gene‐ and potentially age‐dependent. Such insights will increase our understanding of hematopoiesis, clonal evolution, and progression toward malignancy. Ultimately, this knowledge has the potential to inform strategies for risk assessment and selection of HCT donors, paving the way for improved clinical outcomes.

### Limitations of the study

Since no residual graft material was available for LTHIT069, we could not validate the presence or size of the *DNMT3A‐*mutant clones in the graft at the time of HCT for this participant. Yet, based on our findings in the other grafts, as well as previous population studies on CH,^5^, ^6^, ^15^, ^61^ including young individuals, the presence of *DNMT3A*‐mutant clones at detectable levels in this graft from a young individual is highly unlikely. In addition, whereas the selection of *DNMT3A*‐mutant and wildtype HSPCs from the same counterparts allowed the comparison between HSPCs part of a CH clone and their matched counterparts, our targeted approach did not allow inference of the clonal diversity of HSPCs in the phylogenies of the HCT recipients. Finally, we studied a limited number of HCT recipients, including a single cord blood donor. Additional research in larger cohorts, or combining donor material with highly sensitive sequencing technologies, is required to determine the frequency of driver acquisition early in the life or prenatal development of the donor prior to HCT.

## AUTHOR CONTRIBUTIONS

**Lucca L. M. Derks**: Conceptualization; data curation; formal analysis; methodology; software; writing—original draft; writing—review and editing. **Konradin F. Müskens**: Conceptualization; data curation; funding acquisition; methodology; writing—original draft; writing—review and editing. **Markus J. van Roosmalen**: Data curation; methodology; software; writing—review and editing. **Aniek O. de Graaf**: Investigation; resources; writing—review and editing. **Nina Epskamp**: Writing—review and editing; investigation. **Laurianne Trabut**: Investigation; writing—review and editing. **Rico Hagelaar**: Methodology; resources; writing—review and editing; software. **Joop H. Jansen**: Methodology; resources; writing—review and editing; supervision. **Caroline A. Lindemans**: Data curation; writing—review and editing; supervision. **Maaike G. J.M. van Bergen**: Methodology; investigation; resources; writing—review and editing. **Ruben van Boxtel**: Conceptualization; supervision; methodology; funding acquisition; writing—review and editing. **Mirjam E. Belderbos**: Conceptualization; supervision; methodology; funding acquisition; writing—review and editing.

## CONFLICT OF INTEREST STATEMENT

The authors declare no conflicts of interest.

## ETHICS STATEMENT

All participants provided written informed consent. This study was approved by the local ethics committee in accordance with the Declaration of Helsinki (NedMec NL77721.041.21).

## FUNDING

This study was financially supported by a VENI grant of the Netherlands Organization for Scientific Research (grant number VI.Veni.202.021 to M.E. Belderbos), a physician scientist grant of the European Hematology Association (to M.E. Belderbos), a John Hansen research grant from the DKMS Foundation, a Stichting Kinderen Kankervrij project grant (project number 418), and an ERC Consolidator grant from the European Research Council (ERC; no. 864499 to R. van Boxtel). Additionally, this project was supported by the Oncode Institute, funding L.L.M. Derks, M.J. van Roosmalen, L. Trabut, R. Hagelaar, and R. van Boxtel. This research was supported by The New York Stem Cell Foundation. R. van Boxtel is a New York Stem Cell Foundation–Robertson Investigator.

## Supporting information

20250908 Supplementary materials.

## Data Availability

The data that support the findings of this study are openly available in the European Genome‐phenome Archive at https://ega-archive.org/dacs/EGAC00001001864, reference number EGAS50000000919. All WGS data that were generated in this study have been deposited in the European Genome‐phenome Archive (EGA), under accession code (EGAS50000000919). Due to privacy laws, the raw sequencing data are under restricted access, which can be obtained via the Princess Máxima Data Access Committee (https://ega-archive.org/dacs/EGAC00001001864). The processed data, containing only somatic variants, and other data needed to reproduce the figures, are available at the Mendeley Database (preview available: https://data.mendeley.com/preview/ysvds7rgbw?a =b6094db4‐96df‐493f‐aa87‐6a3916e0e323). The variant calling and germline filtering pipelines are available at https://github.com/ToolsVanBox/ASAP. A wrapper for the use of CellPhy as described is available at https://github.com/ToolsVanBox/CellPhyWrapper. All other scripts necessary to reproduce the analyses of this manuscript are available at the Mendeley Database.
