# Correction to “Posttransplantation clonal dynamics of hematopoietic stem cells carrying prenatal and early‐life *DNMT3A* mutations”

**DOI:** 10.1002/hem3.70365

**Published:** 2026-04-28

Derks LLM, Müskens KF, van Roosmalen MJ, et al. Posttransplantation clonal dynamics of hematopoietic stem cells carrying prenatal and early‐life *DNMT3A* mutations. *HemaSphere*. 2025;9(12):e70262. doi:10.1002/hem3.70262

In the General Information column of Table 1, “Recipient age at study (years)*” should have read as “Recipient age at study (years)^a^” to correspond with the table footnote.

Additionally, in the Data Availability Statement, the URL to the Mendeley database was incorrect and has been corrected to https://data.mendeley.com/datasets/ysvds7rgbw/1 (doi:10.17632/ysvds7rgbw.1).

The original article has been updated. We apologize for this error.

