# Supplementary material for: Posttransplantation clonal dynamics of hematopoietic stem cells carrying prenatal and early‐life DNMT3A mutations
Source: Hemasphere. 2025 Dec 9;9(12):e70262. doi: 10.1002/hem3.70262 (PMC12686829; doi:10.1002/hem3.70262)
Supplement: Supplementary file 1 — 20250908 Supplementary materials. [file HEM3-9-e70262-s001.docx]

**Supplementary Materials to ‘Post-transplantation clonal dynamics of hematopoietic stem cells carrying prenatal and early-life DNMT3A mutations’**

**List of Supplementary Materials**

Figure S1: Growth characteristics of *in vitro* expanded colonies.

Figure S2: Analysis of mutant cell fractions in *in vitro* single cell genotyping.

Figure S3: Mutation accumulation in healthy reference hematopoietic stem and progenitor cells.

Figure S4: Comparison of germline filtering references

Figure S5: Contributions and reconstruction of mutational signatures in HCT recipient HSPCs.

Table S1: CH driver information

**
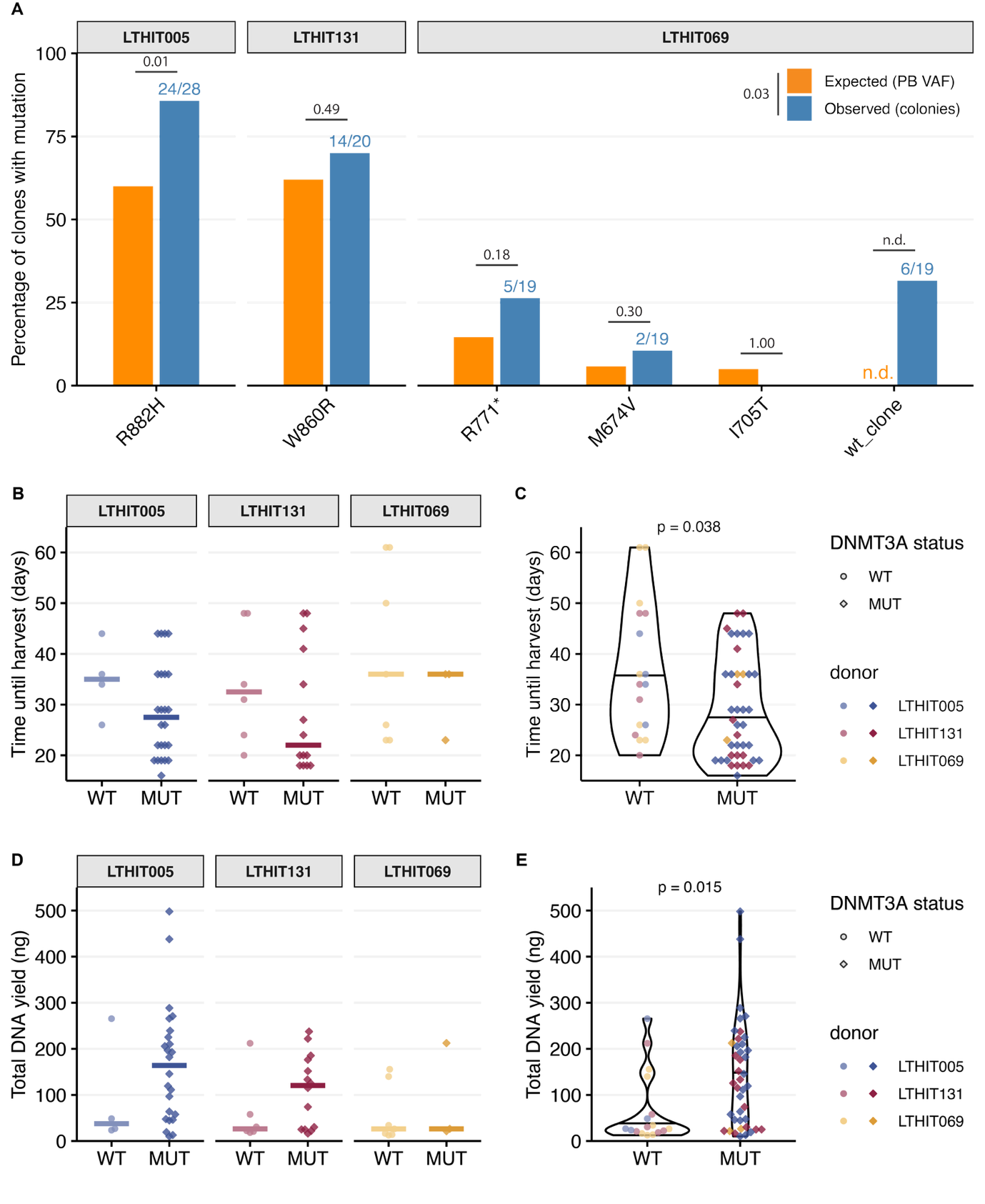
**

**Figure S1: Growth characteristics of *in vitro* expanded colonies.** (**A**) Bar plots depicting the expected percentage of mutant colonies based on the VAF in DNA isolated from whole blood (orange) and the percentage of mutant colonies observed after *in vitro* expansion of sorted HSPCs (blue). No colonies were observed with the p.I705T variant. “wt_clone” refers to the clonal expansion of *DNMT3A*-wildtype HSPCs, observed in phylogenetic analyses. Numbers above the bars indicate the number of mutant colonies and the total number of colonies that were genotyped. P-values were calculated using two-sided binomial tests comparing the observed vs. expected percentage. The p-value next to the legend was calculated from a combined bionomial test, not including the wt_clone. n.d. = not determined. (**B-C**) Quantification of the time from single cell sorting to colony harvest, either per study participant (B) or in total (C). (**D-E**) Quantification of the total DNA yield per colony, either per study participant (D) or in total (E). P-values in B and D were calculated using linear mixed-effects models (lme4 + lmerTest in R), with mutation status as a fixed effect and patient as a random intercept (Satterthwaite’s approximation for degrees of freedom). For LTHIT069, colonies from one plate were harvested prematurely due to a fungal infection and were excluded from these analyses.

**
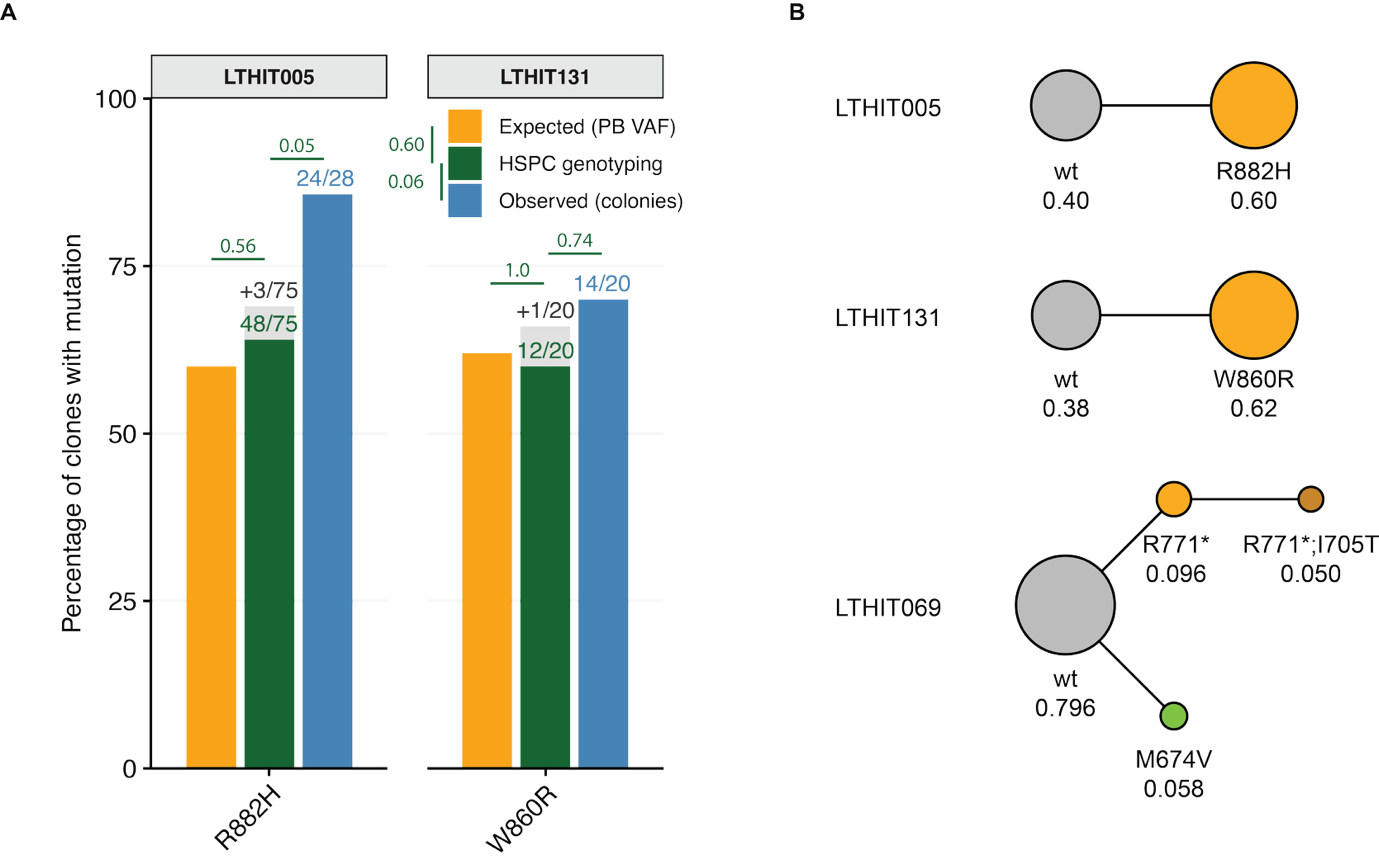
**

**Figure S2: Analysis of mutant cell fractions in *in vitro* single cell genotyping.** (**A**) Bar plots depicting the expected percentage of mutant colonies based on the VAF in DNA isolated from whole blood (orange), the percentage of mutant HSPCs in single-cell genotyping (observed: green, extrapolated false negatives: gray) and the percentage of mutant colonies observed after *in vitro* expansion of sorted HSPCs (blue) for LTHIT005 and LTHIT131. The number of false negative genotyping calls was extrapolated from the frequency of homozygous mutant cells. For LTHIT069, only four HSPCs were successfully genotyped, preventing a reliable estimation of mutation frequencies in the HSPC compartment. Numbers above the bars indicate the number of mutant colonies/HSPCs and the total number of colonies/HSPCs that were genotyped. The p-values next to the legend were calculated from a combined bionomial test. Shown p-values were calculated from two-sided binomial tests, using the observed percentage of mutant cells in single-cell genotyping. P-values using extrapolated values are: R882H HSPC vs. VAF: p = 0.19; R882H HSPC vs. colonies: p = 0.09; W860R HSPC vs. VAF: p = 0.82; W860R HSPC vs. colonies: p = 1.0; combined HSPC vs. VAF: p = 0.17; combined HSPC vs. colonies: p = 0.17. (**B**) Visualisation of clonal composition of the hematopoietic systems of study participants. Circles represent the relative proportions of WT and *DNMT3A*-mutant cells based on the VAF in whole blood DNA, with circle area scaled to proportion. Hierarchy is based on single cell genotyping.


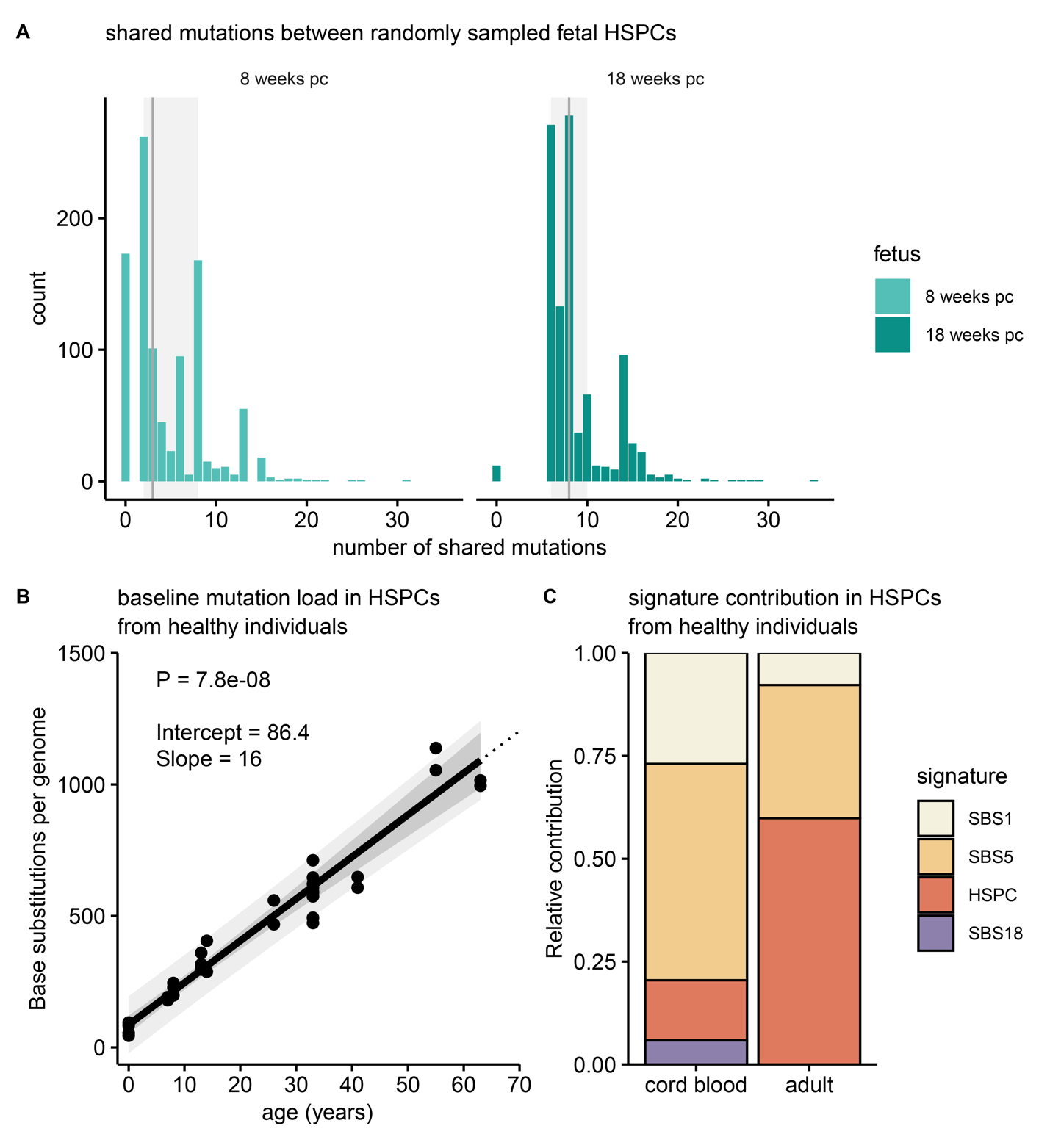


**Figure S3: Mutation accumulation in healthy reference hematopoietic stem and progenitor cells.** (**A**) The number of shared somatic single base substitutions between randomly sampled HSPCs from two fetuses, one representing the ‘sample’ and two the ‘pseudo-bulk’ (Methods)^24^. The vertical grey line indicates the median per fetus and the grey shaded area represents the interquartile range (0.25-0.75) for 1000 iterations of the sampling. pc = post-conception. (**B**) The baseline of linear mutation accumulation of single base substitutions with age in HSPCs from healthy individuals^1,2^, where the data is subsampled to the sequencing depth used in this study (15x). The slope and intercept are derived from a mixed linear model on all HSPC colonies, taking donor dependency into account, which is extrapolated in the dotted line. The age effect was tested by a two-tailed t-test (*p* = 7.8E-8). The shaded areas represent the 95% confidence interval (light grey) and the prediction interval (dark grey) of the mixed model. (**C**) The relative contributions of the single base substitution signatures after bootstrapped refitting (n = 100) extracted through non-negative matrix factorization to HSPCs from healthy individuals^2^. Adult individuals were above 18 years of age.


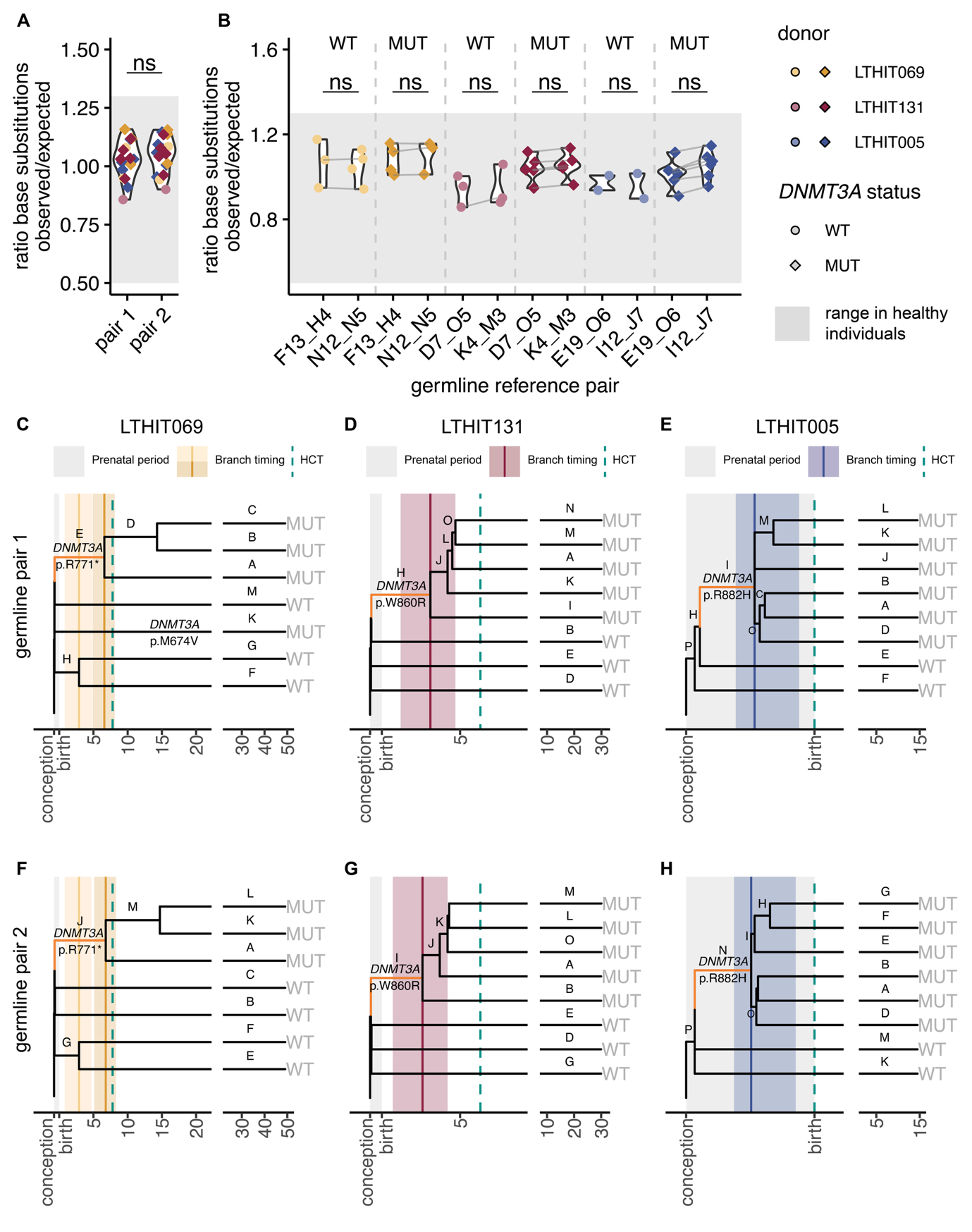


**Figure S4: Comparison of germline filtering references**. (**A**) Somatic autosomal mutation loads of hematopoietic cell transplantation (HCT) recipient HSPCs, normalized to the expected mutation load by hematopoietic age. The mutation loads were determined in two parallel analyses using distinct pairs of HSPCs for germline filtering (pair 1 and pair 2). Samples used in either of the conditions as germline control were excluded, as those samples are unpaired. Comparison of mutation loads was performed by paired Wilcoxon rank sum test (p = 0.413). WT/circles: *DNMT3A* wildtype. MUT/diamonds: *DNMT3A* mutant. (**B**) As in (A), comparing the somatic mutation load within the same HCT recipient and *DNMT3A* status between germline filtering pairs. Testing was performed by unpaired Wilcoxon’s rank sum test and Bonferroni correction (corrected p-values = 1 for all tests). (**C**) Time-scaled phylogenetic tree of single HSPC colonies of LTHIT069, for germline filtering pair 1, including *DNMT3A* wildtype (WT) and mutant (MUT) colonies, obtained after inference by n = 100 bootstrap iterations using a maximum likelihood framework. Vertical dashed line (green) represents the time of HCT. Vertical solid lines and shaded interval (yellow) indicate the time estimate and uncertainty interval of the branchpoint, respectively. (**D**) Similar to (**C**), but for LTHIT131 with time estimates in red. (**E**) Similar to (**C**), but for LTHIT005 with time estimates in blue. (**F-H**) as in (B-D), but for germline filtering pair 2.


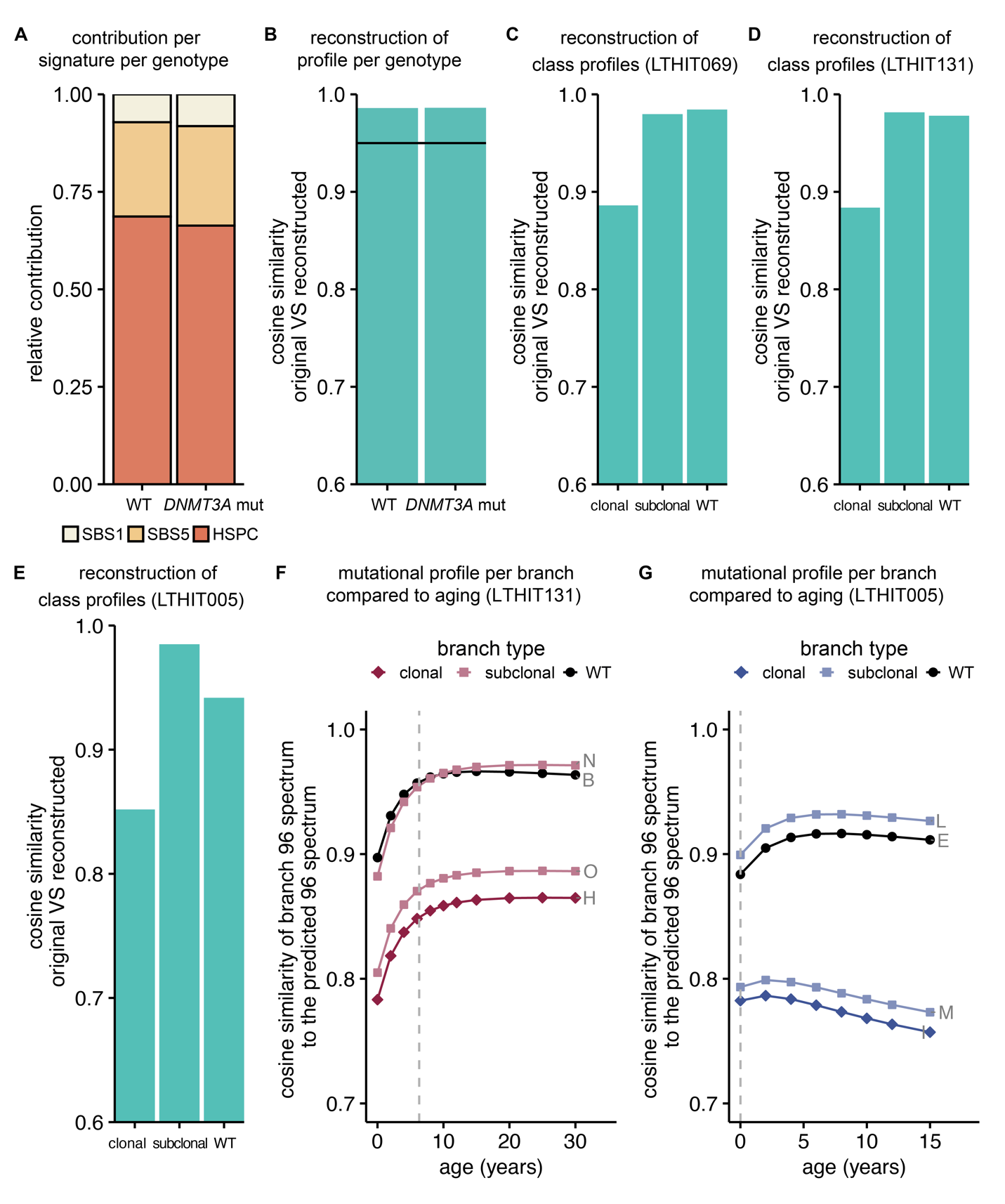


**Figure S5: Contributions and reconstruction of mutational signatures in HCT recipient HSPCs**. (**A**) The contribution per single base substitution signature to all unique somatic mutations identified in recipient HSPCs, obtained after bootstrapped refit (n = 100) of the signatures extracted through non-negative matrix factorization. (**B**) The cosine similarity between the original and reconstructed single base substitution profiles as shown in (A). (**C**) Cosine similarities between the original and reconstructed profiles of the mutation classes of LTHIT069. (**D**) Similar to (C), but for LTHIT131. (**E**) Similar to (C), but for LTHIT005. (**F**) Cosine similarities between the cumulative mutation matrix of a single branch and the predicted mutation matrix at any given age, for the phylogeny or LTHIT131. Representative branches are shown for *DNMT3A* wildtype branches (circles) and subclonal branches (squares). (**G**) Similar to (F), but for LTHIT005.

| **Supplementary Table S1 - CH driver information** | |  |  |  |  |
| --- | --- | --- | --- | --- | --- |
|  |  |  |  |  |  |
| **General information** |  |  |  |  |  |
| Sample name | LTHIT005 | LTHIT131 | LTHIT069 |  |  |
| Recipient Age at study* | 30 | 26 | 42 |  |  |
| HSC age at study | 14.5 | 30 | 49.1 |  |  |
| Sex | Male | Female | Female |  |  |
| **CH driver information** |  |  |  |  |  |
| Mutation number | LTHIT005-1 | LTHIT131-1 | LTHIT069-1 | LTHIT069-2 | LTHIT069-3 |
| Gene | DNMT3A | DNMT3A | DNMT3A | DNMT3A | DNMT3A |
| c.HGVS | 2645G>A | 2578T>C | 2311C>T | 2020A>G | 2114T>C |
| p.HGVS | Arg882His | Trp860Arg | Arg771Ter | Met674Val | Ile705Thr |
| Genomic location (hg19) | chr2: g.25457242 | chr2: g.25458595 | chr2: g.25463182 | chr2: g.25464493 | chr2: g.25463568 |
| Transcript | ENST00000264709 | ENST00000264709 | ENST00000264709 | ENST00000264709 | ENST00000264709 |
| Exon | 23 | 22 | 19 | 17 | 18 |
| Type of mutation (nucleotide) | SBS | SBS | SBS | SBS | SBS |
| Type of mutation (protein) | missense | missense | nonsense | missense | missense |
| **CH measurement at study timepoint** |  |  |  |  |  |
| Material | Whole blood | Whole blood | Whole blood | Whole blood | Whole blood |
| VAF (percentage total) | 30 | 31 | 7.3 | 2.9 | 2.5 |
| Mutant reads (count) | 915 | 836 | 243 | 60 | 94 |
| Total reads at location (count) | 3050 | 2697 | 3329 | 2069 | 3760 |
| **Retrospective CH measurement in graft material** | |  |  |  |  |
| Material | Cord blood | Bone marrow | *N/A* | *N/A* | *N/A* |
| VAF (percentage total) | 0 | 0 | *N/A* | *N/A* | *N/A* |
| Mutant reads (count) | 0 | 0 | *N/A* | *N/A* | *N/A* |
| Total reads at location (count, run 1) | 3690 | 3035 | *N/A* | *N/A* | *N/A* |
| Total reads at location (count, run 2) | 3467 | 2910 | *N/A* | *N/A* | *N/A* |
| **Primers used for genotyping** |  |  |  |  |  |
| Forward primer | AGAACTAAGCAGGCGTCAGA | TGGCCCCTAGACTCCTCTTA | GATGACCCTGTCTTCCCGTG | CTCTCCGGTCAGTTTTCTGC | CAGACTCAGCCGTCCACTTG |
| Reverse primer | TGTCCAACCCTTTTCGCAAG | TGCGAACTCTGCTCACTCA | GTGACAGGTCCTTCAACCCC | TTGCCCTTTACCCTCTCAAGAC | GGAAGACAGGGTCATCGGGA |
| Sequencing primer (colony genotyping) | GGTCCTGCTGTGTGGTTAGA | AAGTCAGGTGGGAAAGGCAG | TCCGAGGTAGGCCTCTAACC | TTCCAGATAGGCCAAACGAGG | CAGACTCAGCCGTCCACTTG |
| Sequencing primer (single-cell genotyping) | GAACTAAGCAGGCGTCAGAGGA | GGAGTCTGCCATGTTGGGAA | same as above | same as above | same as above |
